# Complete occlusion of anterior capsulorhexis after uneventful cataract surgery, treated with YAG laser capsulotomy

**DOI:** 10.1186/s12886-017-0630-0

**Published:** 2017-12-04

**Authors:** Hoon Dong Kim, Jae Min Kim, Jong Jin Jung

**Affiliations:** 1 Department of Ophthalmology, College of Medicine, Soonchunhyang University, Cheonan, Korea; 2 Myung-Gok Eye Research Institute, Department of Ophthalmology, Kim’s Eye Hospital, Konyang University College of Medicine, 136, Yeongsin-ro, Yeongdeungpo-gu, Seoul 07301 Korea

**Keywords:** Anterior capsulorhexis, Capsular contraction syndrome, Fibrotic proliferation, Phacoemulsification, Total occlusion

## Abstract

**Background:**

Capsular contraction syndrome (CCS) has been reported as an uncommon complication after an cataract extraction surgery with intact anterior capsulorhexis. This report is written to present a case of complete occlusion of the anterior capsulorhexis opening after an uneventful cataract surgery, which was treated with non-invasive treatment.

**Case presentation:**

A 69-year-old woman complained of decreased visual acuity in her right eye, which had started 2 months ago. She underwent phacoemulsification with an uneventful anterior capsulorhexis before 3 months. A total occlusion of the anterior capsulorhexis opening with capsular phimosis was identified on slit-lamp biomicroscopy, and a circular anterior capsulotomy using neodymium-doped yttrium aluminum garnet (Nd:YAG) laser was performed immediately. The capsulotomy site remained clear after a couple of years.

**Conclusions:**

It is supposed that proliferation of fibrotic tissue was relatively prominent in this case, rather than the appearance of capsular phimosis. This case can be an uncommon showing a total occlusion of the anterior capsulorhexis opening with prominent fibrotic proliferation pattern after an uneventful cataract surgery. Additionally, the occlusion could be removed with a non-invasive procedure, and was maintained clearly for several years.

## Background

Capsular contraction syndrome (CCS) has been reported as an uncommon complication after an cataract extraction surgery with intact anterior capsulorhexis [1–3]. It is treated by surgical removal or application of laser to the thickened and opaque anterior capsulorhexis site. It is considered that most cases of CCS are associated with an underlying disease showing zonular weakness and chronic inflammation [2, 3]. However, several patients of CCS without any clinical signs of obvious zonular weakness or inflammatory reaction were presented in previous reports [1–4]. Therefore, we would like to report a case of complete occlusion of the anterior capsulorhexis opening after an uneventful cataract surgery, that was treated with neodymium-doped yttrium aluminum garnet (Nd:YAG) laser.

## Case presentation

**Fig. 1:**
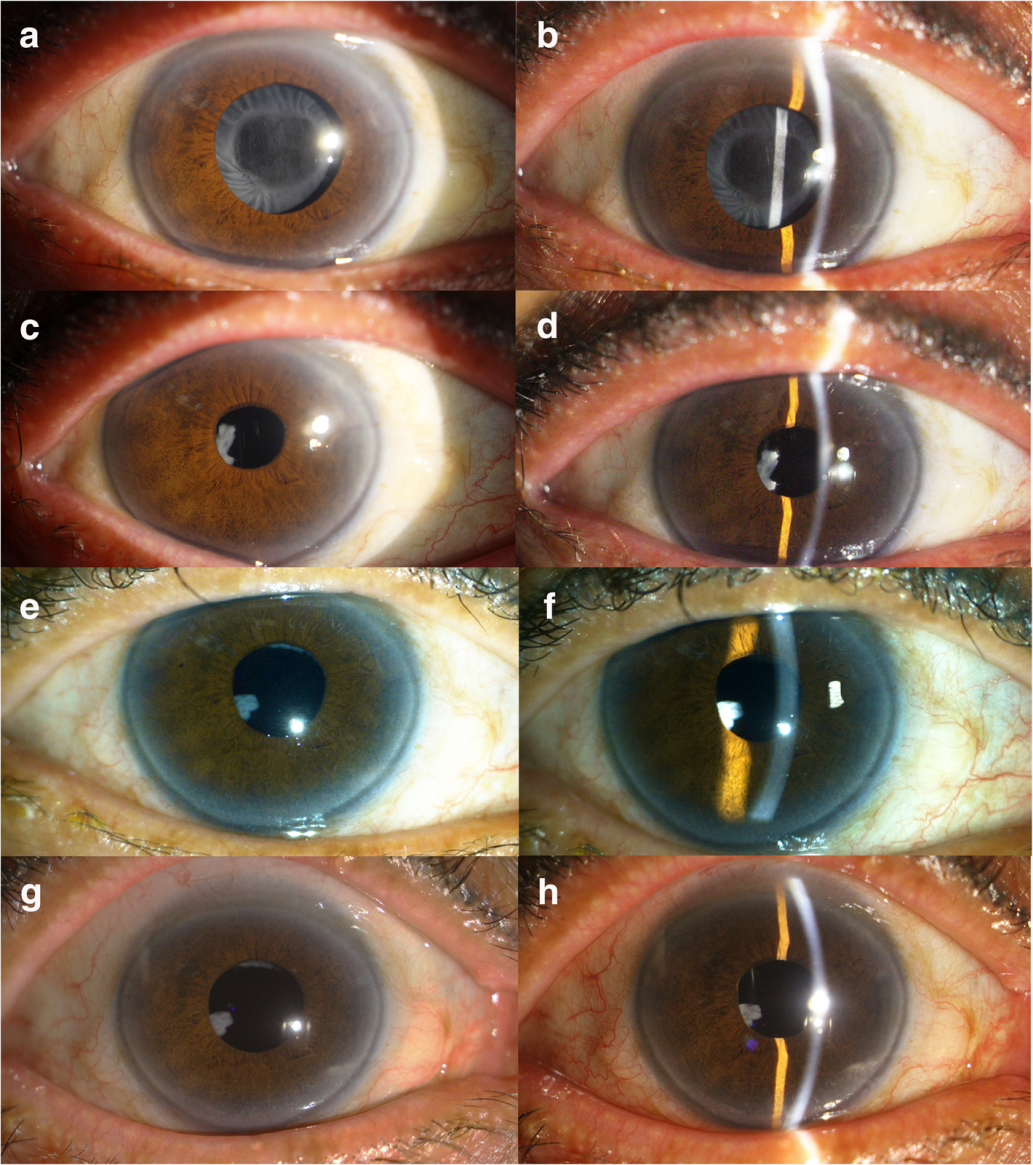
Consecutive findings of silt-lamp biomicroscopy on the right eye. Total occlusion of anterior capsulorhexis opening was identified (**a**, **b**). Cleared anterior capsulorrhexis site followed by anterior capsulotomy using Nd:YAG laser (**c**, **d**). Capsulotomy site remained clearly after 6 months (**e**, **f**) and there was no remarkable change on capsulotomy site after 2 years (**g**, **h**)

A 69-year-old woman visited our clinic, complaining of decreased visual acuity in her right eye, which had started 2 months ago. There was no documented history of any systemic disease or drug intake. Three years ago, she had undergone laser iridotomy in the right eye following an angle-closure attack. Phacoemulsification with an uneventful anterior capsulorhexis was performed. Intraoperatively, the anterior capsulorhexis opening was noted to approximately 5.5 mm in diameter, and clinical features of prominent zonular weakness were not evident. A one-piece aspheric hydrophobic acrylic intraocular lens with an overall diameter of 13.0 mm, optic diameter of 6.0 mm and power of +22.0 diopters was implanted in the bag without any decenteration. The best corrected visual acuity (BCVA) was 20/20 in both eyes postoperatively, and there were no remarkable findings. Four months later, however, her visual acuity was found to have decreased to 20/60 in the right eye. Slit-lamp biomicroscopy revealed a total occlusion of the anterior capsulorhexis opening with capsular phimosis (Fig. 1). Immediately, a circular anterior capsulotomy using Nd:YAG laser was performed. After 1 month, the capsular phimosis had not recurred, and additional findings about zonular weakness were not apparent. The BCVA in the right eye had recovered to 20/20 with no other remarkable findings under slit-lamp biomicroscopy (Fig. 1). The capsulotomy site remained clear after 2 years, and the visual acuity was also unchanged (Fig. 1).

## Discussion and conclusions

CCS is one of the well-known complications of continuous curvilinear capsulorrhexis. It has been described as an exaggerated fibrotic response that can lead to a reduction in the size of the anterior capsulotomy [1, 2]. Thereafter, CCS results in impaired visual function secondary to the opacity in pupillary area. It has been associated with uveitis, pseudoexfoliation, myotonic dystrophy, and retinitis pigmentosa [3]. CCS has also occurred with small capsulorrhexis openings of less than 6 mm diameter, and Acrylic IOL revealed lowest rates [4]. The present case was an unusual, compared with previously reported CCS cases that were treated with Nd:YAG laser radial capsulotomy, because an uneventful surgery without zonular weakness was performed, the patient had no underlying disease except for a history of an angle-closure attack; furthermore, anterior capsulorhexis opening was completely occluded. In addition, proliferation of fibrotic tissue was relatively prominent in this case, rather than the appearance of capsular phimosis. In previous studies, CCS was thought to have been caused by two underlying mechanisms; shrinkage of the capsulorhexis leading to the formation of smaller diameter capsular opening, and the development of a fibrocellular membrane caused by lens epithelial cells closing remaining central opening [5]. Spang et al. noted that complete occluded anterior capsulorhexis opening was filled with proliferated lens epithelial cells under light microscopy, and the cellular elements revealed a positive reaction for actin filament upon immunohistochemical analysis [5]. In our opinion, this is an uncommon case showing a total occlusion of the anterior capsulorhexis opening with prominent fibrotic proliferation pattern after an uneventful cataract surgery. Additionally, the occlusion could be removed with a non-invasive procedure, and was maintained clearly for a couple of years.

## Abbreviations

BCVA: Best corrected visual acuity
CCS: Capsular contraction syndrome
Nd:YAG: Neodymium-doped yttrium aluminum garnet
